# Response to letter from van der Zaag et al

**DOI:** 10.1017/ash.2025.10075

**Published:** 2025-09-15

**Authors:** Christopher Doern, Melissa Whitman, Michelle Doll, Susan Roseff, Alexandra Bryson

**Affiliations:** 1 Department of Pathology, Virginia Commonwealth University Health System, Richmond, VA, USA; 2 Department of Internal Medicine, Virginia Commonwealth University Health System, Richmond, VA, USA

Dear editor,

We thank Dr van der Zaag et al for their thoughtful commentary on our manuscript “Blood culture bottle shortage mitigation efforts: analysis of impact on ordering and patient impact,” and we thank you for the opportunity to respond. We agree with nearly all points made in their letter. The analysis we published included the first 21 days of postintervention data. This time frame is too short, and the methods are inadequate to definitively assess the impact these interventions had on patient outcomes. Long-term patient follow-up was beyond the scope of this work, but we agree that our conclusions should be considered preliminary and warrant deeper investigation of their impact beyond the immediate postintervention period. We are currently conducting an investigation to include an assessment of blood culture positivity rates as well as many of the outcome measures mentioned by van der Zaag et al including late diagnosis of bacteremia after an initial single negative blood culture, readmission for infection/sepsis, and mortality. Of particular importance, there will be an assessment of sepsis measures before, during, and after the shortage, especially sepsis-related mortality, and time to first blood culture in patients who were ultimately diagnosed with sepsis.

Our hope in publishing the early assessment of our mitigation efforts was to provide some insight into how institutions might manage the shortage. Given the urgency of the matter and the concern that the shortage would progress to an inability to perform any blood cultures, we carefully considered the best methods to actively monitor the situation and ensure that standards of care were preserved. It is worth noting that the EMR algorithm we used to promote blood culture stewardship is based on published data suggesting little additional diagnostic value of positive blood cultures in the setting of active treatment for source infections like cellulitis, pneumonia, and urinary tract infections, as well as prospective studies that have robustly evaluated for adverse patient outcomes after implementation of diagnostic stewardship interventions and finding no evidence of patient safety compromise.^1,2^ Using this algorithm, practice should have changed only for patients at low or no risk of bacteremia. Although randomized controlled trials (RCTs) would be best to definitively address patient safety concerns, that was not our goal in this report. Instead, our focus was on the immediate behavioral impact of the intervention on our ordering providers, and the primary, real-time metric available to us was changes in percent positivity. Although this is not a metric of patient outcome, it is an important measure of how blood culture utilization changed in response to our mitigation efforts. Overall, our positivity rates increased during our study period and indeed over the remainder of the shortage (data not published). We were especially encouraged by the fact that in our highest volume location, and the location in which most of our bacteremias are diagnosed, the emergency department (ED), we saw excellent correlation between the decrease in test volume and the concomitant increase in blood culture positivity. The correlation one would hope to observe if the denominator of negative cultures were reduced by our interventions. The average blood culture positivity rate in our ED from June 2022 to May 2023 was 10.6% over 24,224 blood cultures. During the blood culture shortage (intervention period) our ED blood culture utilization decreased by 40.8%. If one were to eliminate 40.8% of the 24,224 ED blood cultures and project the positivity rate if only negative blood cultures were reduced (our goal), the resulting positivity rate would be 16.7%. During our actual intervention period, the blood culture positivity rate in our ED was 19.7%, 3.2% greater than projected. We recognize that there are far too many variables in this analysis to feel comfortable concluding anything meaningful, but it does raise the intriguing possibility that our interventions not only reduced unnecessary blood culture orders but may have actually improved our ability to diagnose bacteremia, something we will assess in our ongoing follow-up study.

In closing, we agree with van der Zaag et al and fully support performing RCTs and outcome-based studies to evaluate practice changes when feasible. The data from our publication was submitted in the midst of a crisis in hopes that our experiences could help others mitigate the crisis. Obviously, in this setting, an RCT was not possible. Although imperfect, we used a metric that we believe could be used to provide some assurance that their interventions were not significantly harming patient care. Further, we believe that in many cases, if not most, RCTs are not possible, and the absence of RCTs should not prohibit the introduction of evidence-based decision support, provided facilities monitor for evidence of unintended consequences. It is well-documented that inappropriate utilization of laboratory testing is pervasive throughout medicine and has begotten efforts such as the “Choosing Wisely” campaign along with an explosion of interest in Diagnostic Stewardship Programs.^1–4^ We commend van der Zaag et al for developing a modeling system that can assess the impact of decision support tools and hope that their efforts will set a precedent for our field to more easily assess the impact of diagnostic stewardship interventions.
